# Acidosis Activation of the Proton-Sensing GPR4 Receptor Stimulates Vascular Endothelial Cell Inflammatory Responses Revealed by Transcriptome Analysis

**DOI:** 10.1371/journal.pone.0061991

**Published:** 2013-04-16

**Authors:** Lixue Dong, Zhigang Li, Nancy R. Leffler, Adam S. Asch, Jen-Tsan Chi, Li V. Yang

**Affiliations:** 1 Department of Oncology, Brody School of Medicine, East Carolina University, Greenville, North Carolina, United States of America; 2 Department of Internal Medicine, Brody School of Medicine, East Carolina University, Greenville, North Carolina, United States of America; 3 Department of Anatomy and Cell Biology, Brody School of Medicine, East Carolina University, Greenville, North Carolina, United States of America; 4 UNC Lineberger Comprehensive Cancer Center, Chapel Hill, North Carolina, United States of America; 5 Institute for Genome Sciences and Policy, Duke University, Durham, North Carolina, United States of America; 6 Department of Molecular Genetics and Microbiology, Duke University, Durham, North Carolina, United States of America; University of York, United Kingdom

## Abstract

Acidic tissue microenvironment commonly exists in inflammatory diseases, tumors, ischemic organs, sickle cell disease, and many other pathological conditions due to hypoxia, glycolytic cell metabolism and deficient blood perfusion. However, the molecular mechanisms by which cells sense and respond to the acidic microenvironment are not well understood. GPR4 is a proton-sensing receptor expressed in endothelial cells and other cell types. The receptor is fully activated by acidic extracellular pH but exhibits lesser activity at the physiological pH 7.4 and minimal activity at more alkaline pH. To delineate the function and signaling pathways of GPR4 activation by acidosis in endothelial cells, we compared the global gene expression of the acidosis response in primary human umbilical vein endothelial cells (HUVEC) with varying level of GPR4. The results demonstrated that acidosis activation of GPR4 in HUVEC substantially increased the expression of a number of inflammatory genes such as chemokines, cytokines, adhesion molecules, NF-κB pathway genes, and prostaglandin-endoperoxidase synthase 2 (PTGS2 or COX-2) and stress response genes such as ATF3 and DDIT3 (CHOP). Similar GPR4-mediated acidosis induction of the inflammatory genes was also noted in other types of endothelial cells including human lung microvascular endothelial cells and pulmonary artery endothelial cells. Further analyses indicated that the NF-κB pathway was important for the acidosis/GPR4-induced inflammatory gene expression. Moreover, acidosis activation of GPR4 increased the adhesion of HUVEC to U937 monocytic cells under a flow condition. Importantly, treatment with a recently identified GPR4 antagonist significantly reduced the acidosis/GPR4-mediated endothelial cell inflammatory response. Taken together, these results show that activation of GPR4 by acidosis stimulates the expression of a wide range of inflammatory genes in endothelial cells. Such inflammatory response can be suppressed by GPR4 small molecule inhibitors and hold potential therapeutic value.

## Introduction

The induction of vascular endothelial cell inflammatory responses is important for various pathophysiological conditions [1], [2], [3], [4]. For instance, the increased adhesiveness and inflammatory cytokine production of endothelial cells play pivotal roles in the recruitment of leukocytes to inflammatory sites. In this process, leukocytes first adhere to the activated (inflamed) endothelial cells, become stimulated, and then transmigrate through vascular endothelium into inflammatory tissues. The increased production of vascular adhesion molecules, chemokines and cytokines in endothelial cells is critical for the endothelium-leukocyte interaction [2]. Moreover, leukocyte infiltration is commonly observed in solid tumors and is important for cancer progression and tumor immunity [1]. Endothelial cell inflammatory responses also promote the adherence of blood cells to vessel wall, which may lead to vaso-occlusion and tissue ischemia as observed in stroke, myocardial infarction, sickle cell disease, and many other diseases [3], [4]. It is, therefore, of significant importance to identify factors and molecular pathways that regulate endothelial cell inflammatory responses in order to devise new approaches to treat inflammation and vaso-occlusion.

A myriad of studies show that localized interstitial acidosis is a biochemical hallmark in inflammatory tissues, ischemic organs, and solid tumors [5], [6], [7], [8], [9], [10], [11]. The acidification of local tissues can be caused by dysregulated cell metabolism and/or defective blood perfusion to remove acidic metabolic byproducts. Using microelectrode or non-invasive imaging approaches, an interstitial tissue pH below 7.0, and sometimes even below 6.0, has been observed in stroke, myocardial infarction, tumors, and inflammatory diseases such as asthma and arthritis [6], [8], [10], [12]. Interstitial acidosis has been shown to cause tissue injury and aggravate disease progression [6], [8], [10]. Nevertheless, the effects of acidosis on vascular endothelial cells and the molecular pathways by which endothelial cells respond to acidosis are largely unknown.

Recent studies suggest that the proton-sensing receptor GPR4 is a functional pH sensor for endothelial cells to perceive acidic extracellular pH [13], [14], [15]. Our previous results show that activation of GPR4 by either isocapnic acidosis or hypercapnic acidosis (due to carbon dioxide accumulation) increases the adhesiveness of HUVECs through the cAMP/Epac pathway [13]. In the current study, we have used the whole-genome transcriptomic analyses to assess the effects of acidosis activation of GPR4 in human vascular endothelial cells. The results show that activation of GPR4 by acidic pH augments the overall acidosis response and particularly stimulates the expression of a wide range of inflammatory genes. Importantly, treatment with a small molecule inhibitor of GPR4 abolishes the acidosis/GPR4-mediated endothelial inflammatory response, suggesting that targeting GPR4 may be exploited as a potential approach to inhibit inflammation and vaso-occlusion in various pathological conditions.

## Results

### The global gene expression of GPR4-mediated acidosis response in HUVEC

**Figure 1:**
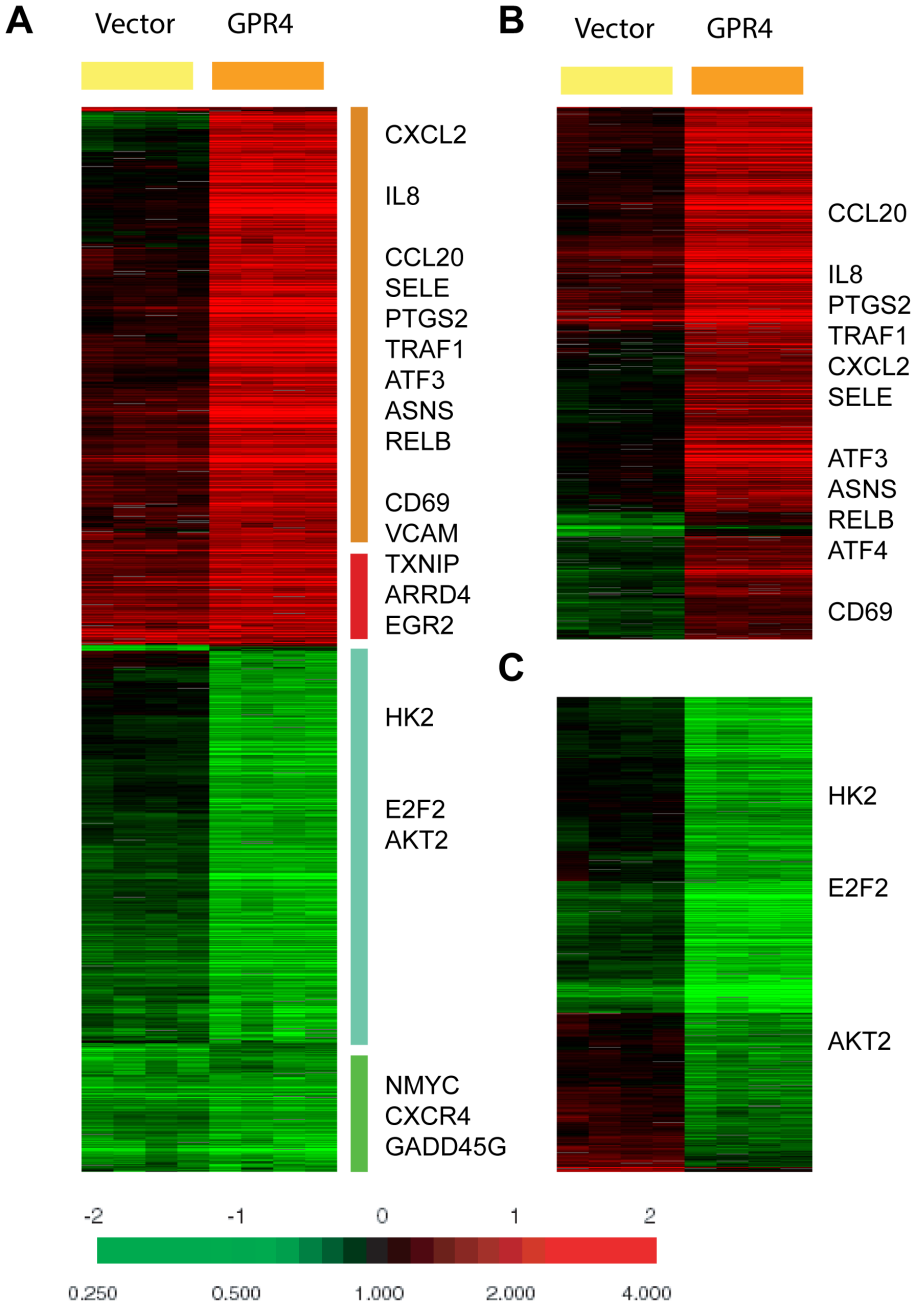
The global gene expression of acidosis response of HUVEC. (A) The gene expression response of HUVECs transduced with the control vector or the GPR4 expression construct is shown. 1208 probes were selected by the criteria of at least three observations with at least three fold changes and arranged by hierarchical clustering. Clusters of genes which are induced or repressed in a GPR4-depedent or -independent fashion are shown with the names of selected genes. (B, C) The gene expression responses of the probes selected by SAM to be 941 GPR4-induced (B) or 679 GPR4-repressed (C) are depicted with the names of selected genes shown.

To examine the effects of acidosis/GPR4 signaling on gene expression at the whole-genome level, we used microarrays to compare the global gene expression response to acidosis in HUVECs which had been stably transduced with either empty vector (HUVEC/Vector cells) or human GPR4 cDNA (HUVEC/GPR4 cells). The GPR4 mRNA level in HUVEC/GPR4 cells was about 10-fold higher than that in HUVEC/Vector cells as previously reported [13]. The overexpression of GPR4 is potentially relevant as GPR4 expression can be up-regulated by stimuli such as TNF-α and H_2_O_2_ in endothelial cells [16]. Previous studies demonstrate that GPR4 has high receptor activity around pH 6.4 (400 nM H^+^) and minimal activity around pH 8.4 (4 nM H^+^) [13], [15], [17]. Therefore, HUVEC/Vector and HUVEC/GPR4 cells in four replicates were treated with pH 6.4 for 5 hours to activate GPR4, and treated with pH 8.4 for 5 hours to serve as negative controls. The same amount of Cy5-labeled sample cRNA (from pH 6.4-treated cells) and Cy3-labeled control cRNA (from pH 8.4-treated cells) of corresponding pairs of HUVEC was hybridized with the Agilent Whole Genome Microarray Chip. Since this is a dual-color array, the Cy5/Cy3 ratio of each gene directly indicates the change in the expression in response to acidosis. The normalized acidosis response were filtered using the criteria of presence in >80% arrays with absolute variations of >3 fold in at least 3 arrays to select 1208 genes (Table S2). These selected genes were then arranged by hierarchical clustering and revealed that the overall acidosis response in HUVEC was greatly enhanced by GPR4 overexpression (Fig. 1A).

Among the acidosis-induced genes, there were genes whose induction was either dependent or independent of the varying GPR4 levels. A few genes previously reported to be induced by acidosis in breast cancer cells [18], including TXNIP, ARRDC4 and EGR2, were also noted to be induced in both HUVEC/Vector and HUVEC/GPR4 cells (Fig. 1A). However, some acidosis-induced genes were either increased by several-fold or unchanged in the control HUVEC/Vector cells but significantly further induced in HUVEC/GPR4 cells (Fig. 1A). These genes include many inflammatory genes (e.g. CXCL2, CCL20, IL8, TRAF1, RELB, CD69, SELE, VCAM1 and PTGS2) and stress response genes (e.g. ATF3, ATF4, DDIT3 and ASNS). Similarly, a small set of genes were repressed in both HUVEC/Vector and HUVEC/GPR4 cells, including N-myc, GADD45G, and CXCR4. The reduction of some genes, such as E2F2, HK2 (hexokinase 2), and AKT2, however, were greatly enhanced by a higher level of GPR4 in HUVEC/GPR4 cells. The repression of AKT2 and HK2 is consistent with a previous report on the ability of acidosis to repress AKT and glycolysis in breast cancer cells [19].

To formally define the gene expression program regulated by GPR4, we used SAM (Significance Analysis of Microarrays) [20] to identify the subsets of genes whose expression was significantly altered by the varying level of GPR4 between HUVEC/Vector cells and HUVEC/GPR4 cells. We selected 941 and 679 genes which were either induced or repressed, respectively, by GPR4 overexpression at 0% false discovery rate (Table S3 and Table S4). When the expression of the SAM selected gene lists were examined in the context of HUVEC acidosis response, we found that the expression of most genes induced by GPR4 overexpression was increased by acidic pH (Fig. 1B). Among the 941 induced probes selected by SAM, 908 (at *P*<0.05) and 840 (at *P*<0.01) probes were statistically higher in the GPR4-overexpressing cells, respectively. Moreover, the expression of most genes repressed by GPR4 overexpression was decreased by acidic pH (Fig. 1C). Among the 679 repressed probes selected by SAM, 670 (at *P*<0.05) and 636 (at *P*<0.01) probes were statistically lower in the GPR4-overexpressing cells, respectively. The Gene Ontology (GO) enrichment was performed for the genes that were induced by GPR4 overexpression in HUVEC/GPR4 cells using the GATHER program [21], and found that immune, defense and inflammatory responses were significantly enriched (Table S5). In contrast, the DNA-dependent transcription and nucleotide metabolism were enriched for the genes that were repressed by GPR4 overexpression (Table S6). These results were consistent with the GPR4-dependent induction of the inflammatory response (Table 1). Together, these data show a convincing and critical role of GPR4 in the acidosis gene expression response of HUVEC.

**Table 1:** A partial list of acidosis/GPR4-induced inflammatory genes by microarray analysis.

Gene ID	Gene Symbol	Fold Changes (average)	
		Vector* (pH 6.4/pH 8.4)	GPR4* (pH 6.4/pH 8.4)
3576	IL8	6.9	50.4
6354	CCL7	4.4	16.2
6372	CXCL6	3.8	15.0
2920	CXCL2	3.2	103.7
2919	CXCL1	2.9	40.0
1437	CSF2	2.9	9.3
6376	CX3CL1	2.8	17.3
2921	CXCL3	2.4	43.6
6364	CCL20	2.3	78.2
6347	CCL2	2.1	25.8
6352	CCL5	1.6	16.8
3552	IL1A	1.2	19.1
7412	VCAM1	6.5	65.9
6401	SELE	2.5	125.3
3383	ICAM1	1.3	7.7
3604	TNFRSF9	1.6	5.5
79931	TNIP3	1.5	21.3
7128	TNFAIP3	1.4	31.2
7127	TNFAIP2	1.4	10.5
5971	RELB	1.4	6.4
25816	TNFAIP8	1.3	9.7
970	TNFSF7	1.3	8.0
4792	NFKBIA	1.2	8.9
7185	TRAF1	1.1	8.2
64332	NFKBIZ	1.0	6.2
1958	EGR1	12.6	32.7
1959	EGR2	14.6	14.0
1960	EGR3	1.5	6.1
10628	TXNIP	6.6	6.3
5743	PTGS2	1.3	17.8

* Vector: HUVEC/Vector cells, pH 6.4 vs. 8.4; GPR4: HUVEC/GPR4 cells, pH 6.4 vs. 8.4.

### The GPR4-mediated induction of inflammatory genes in HUVEC

The inflammatory genes with substantial up-regulation by the acidosis/GPR4 signaling include: chemokines and cytokines (CXCL1, CXCL2, CXCL3, CXCL6, CX3CL1, CCL2, CCL5, CCL7, CCL20, CSF2, IL1A, IL8), adhesion molecules (E-selectin (SELE), VCAM1, ICAM1), several genes involved in the TNF pathway (TNFRSF9, TNFSF7, TRAF1, TNFAIP2, TNFAIP3, TNFAIP8, TNIP3) and the NF-κB pathway (NFKB1, NFKB2, RELB, NFKBIA, NFKBIZ), the inflammatory enzyme prostaglandin-endoperoxidase synthase PTGS2 (COX-2), transcription factors early growth response (EGR) 1, 2 and 3, and TXNIP.

We then further assessed the GPR4-mediated induction of the inflammatory genes which represent a prominent signature in our microarray dataset. For the majority of the inflammatory genes, GPR4 overexpression further augmented their up-regulation by acidic pH in HUVECs (Table 1). For instance, the expression of IL8 was increased by 6.9 fold in HUVEC/Vector cells and 50.4 fold in HUVEC/GPR4 cells by pH 6.4 in comparison to pH 8.4. The results indicate that these genes are directly induced by acidosis/GPR4 signaling. For few genes, such as EGR2 and TXNIP, the fold of up-regulation responding to acidosis was similar in HUVEC/Vector and HUVEC/GPR4 cells (Table 1), suggesting that the induction of these genes is through GPR4-independent mechanisms.

### Validation of the differential gene expression identified by the microarray analysis

**Figure 2:**
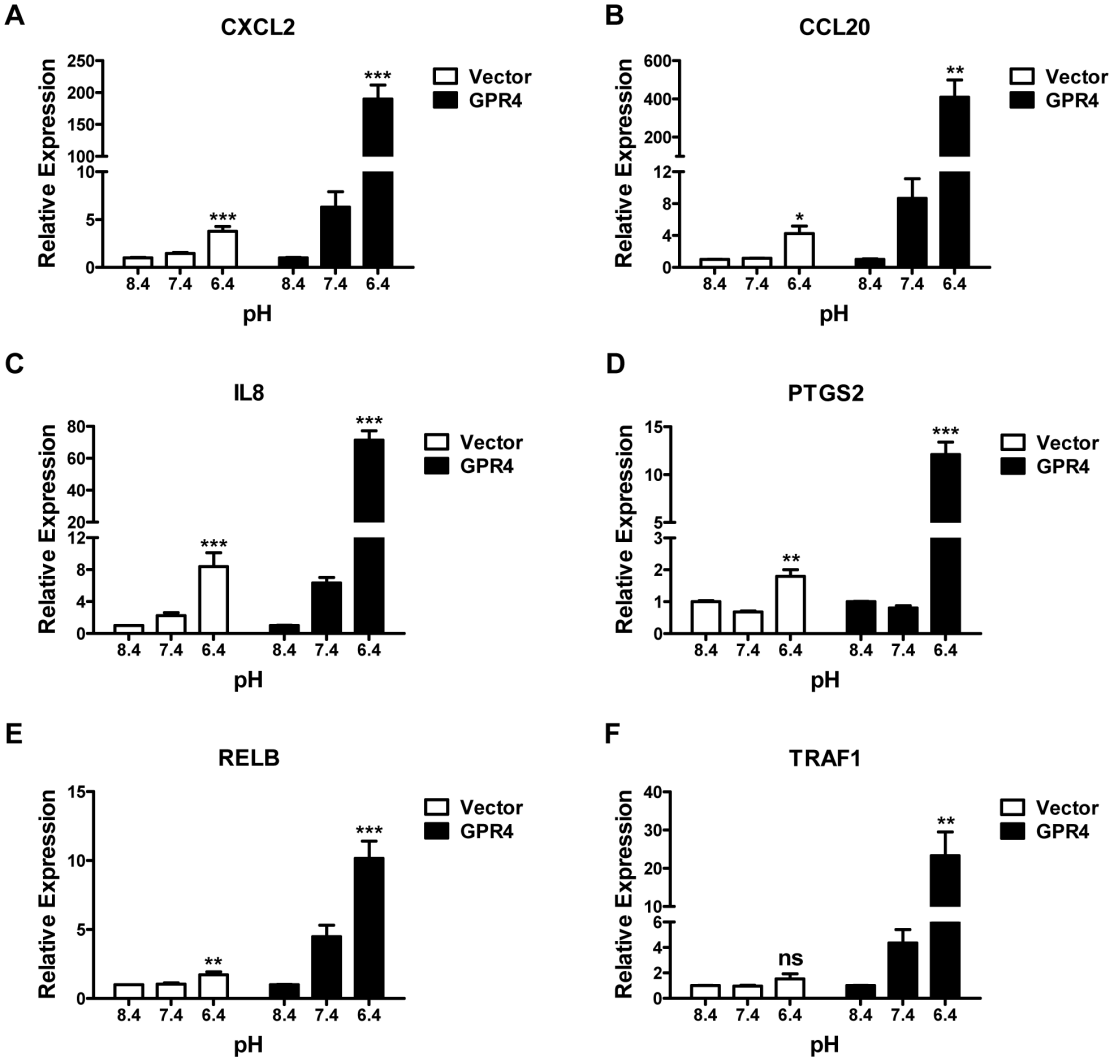
Validation of microarray by real-time RT-PCR. HUVECs transduced with the control vector (Vector, white bars), or the GPR4 expression construct (GPR4, dark bars) were treated with EGM-2/HEM media at pH 8.4, 7.4, or 6.4 for 5 h. Total RNA was isolated and cDNA was synthesized. Real-time RT-PCR quantification of mRNA levels of CXCL2 (A), CCL20 (B), IL8 (C), PTGS2 (D), RELB (E) and TRAF1 (F) was performed. Ct values were normalized to the housekeeping gene GAPDH. The expression level of the target gene in HUVEC/Vector or HUVEC/GPR4 cells at pH 8.4 was set as 1. Error bars indicate the mean ± SEM. *, *P*<0.05; **, *P*<0.01; ***, *P*<0.001; *ns*, not significant (*P*>0.05); compared with the pH 8.4 groups. The results shown are the average of at least two biological repeats.

**Figure 3:**
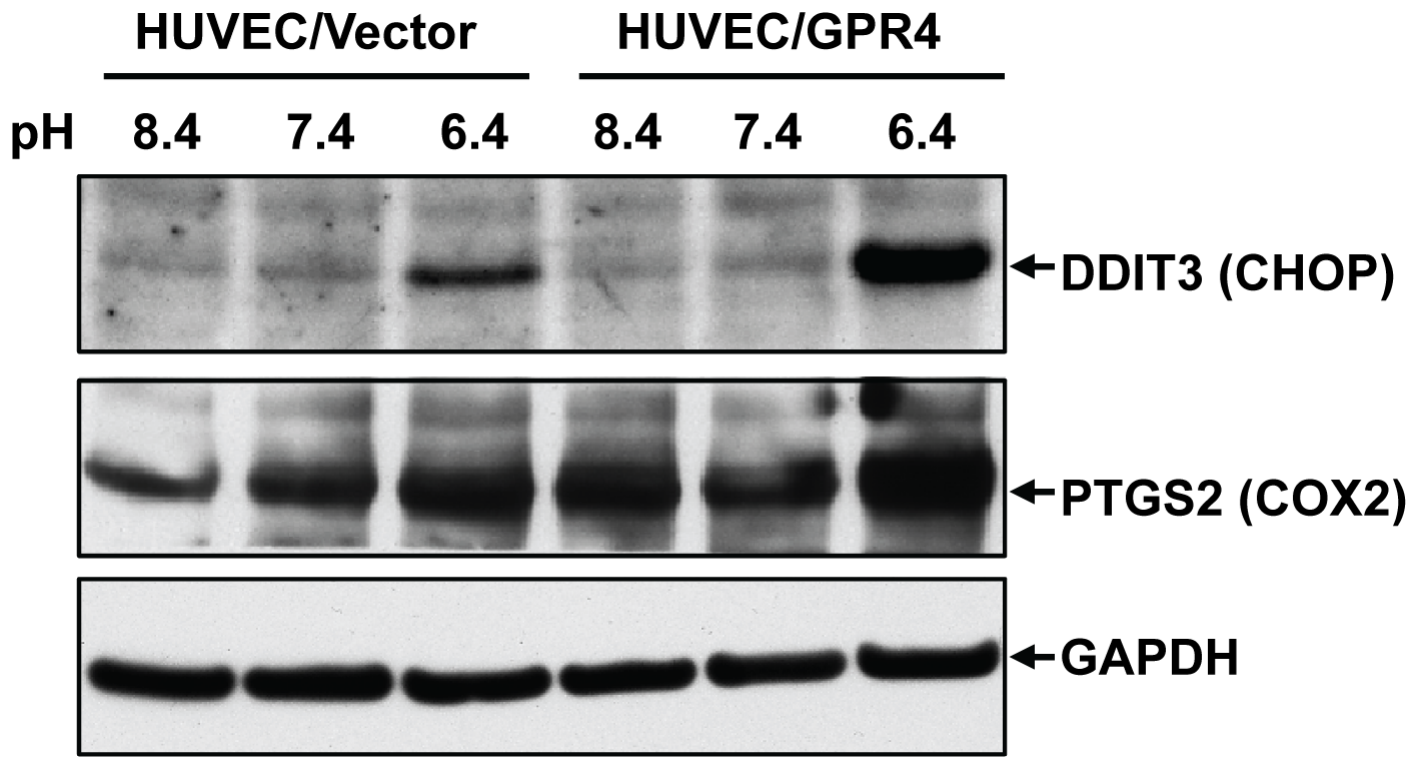
Validation of gene expression at the protein level by Western blotting. HUVEC/Vector and HUVEC/GPR4 cells were treated with EGM-2/HEM media at pH 8.4, 7.4, or 6.4 for 5 h. Cells were then lysed with RIPA buffer, and total proteins were separated by electrophoresis and transferred to nitrocellulose membrane. Protein expression of DDIT3 (CHOP) and PTGS2 (COX-2) was detected using the specific antibodies. The target bands are indicated by an arrow. Western blot of GAPDH serves as a loading control. The results shown are representative of three experiments.

TapMan real-time RT-PCR was performed to confirm the expression of a number of genes induced by acidosis/GPR4. Total RNA was isolated from HUVEC/Vector and HUVEC/GPR4 cells that were treated with pH 6.4 (400 nM H^+^), pH 7.4 (40 nM H^+^), or pH 8.4 (4 nM H^+^) for 5 hours. After normalized to the internal control GAPDH gene, the fold change of gene expression was calculated by the 2^−ΔΔCt^ method [22]. Normalization to two other housekeeping genes, β-actin and 18S rRNA, gave similar results. We have confirmed the expression of 18 genes. The representative real-time RT-PCR results of 6 genes are shown in Fig. 2. Table S7 includes the quantitative gene expression changes of all 18 genes including CXCL2, CCL20, VCAM1, E-selectin, ICAM1, CD69, IL8, IL1A, PTGS2 (COX-2), RELB, TRAF1, EGR1, EGR2, EGR3, DDIT3 (CHOP), FOXF1, ATF3 and KLF9 (Table S7). Overall, the real-time RT-PCR results were consistent with the microarray results. Compared to the physiological pH 7.4 and basic pH 8.4, the acidic pH 6.4 increased the expression of these genes in HUVECs. Moreover, the overexpression of GPR4 further stimulated the expression of the majority of these genes in response to acidosis in HUVEC/GPR4 cells, suggesting that these genes are regulated by acidosis/GPR4 signaling. To further validate the gene expression at the protein level, we performed Western blotting to examine the expression of DDIT3 (CHOP) and PTGS2 (COX-2) in HUVEC. The results showed that acidic pH treatment increased the protein expression of DDIT3 and PTGS2 in HUVEC/Vector cells, which was further increased in HUVEC/GPR4 cells (Fig. 3). The induced expression of PTGS2 (COX-2) by acidic pH is concordant with a previous study using bovine corneal endothelial cells [23].

**Figure 4:**
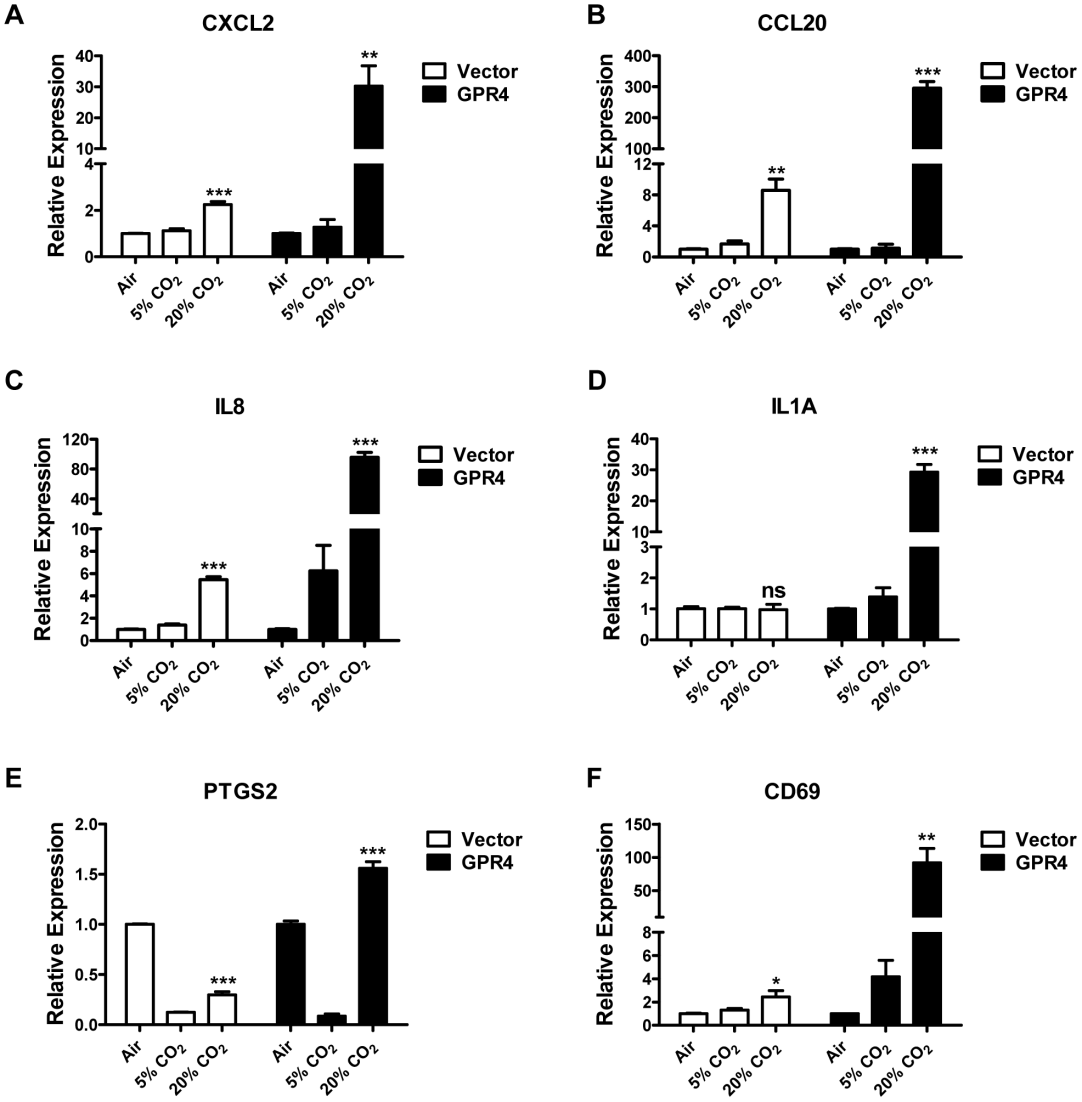
Hypercapnic acidosis activation of GPR4 increases the expression of inflammatory genes in HUVEC. HUVECs transduced with the control vector (Vector, white bars), or GPR4 expression construct (GPR4, dark bars) were treated for 5 h with EGM-2 media buffered with ambient air, 5% CO_2_ or 20% CO_2_. Real-time RT-PCR quantification of mRNA levels of CXCL2 (A), CCL20 (B), IL8 (C), IL1A (D), PTGS2 (E), and CD69 (F) was performed. Ct values were normalized to the housekeeping gene GAPDH. The expression level of the target genes in HUVEC/Vector or HUVEC/GPR4 cells treated with ambient air-buffered EGM-2 medium was set as 1. Error bars indicate the mean ± SEM. *, *P*<0.05; **, *P*<0.01; ***, *P*<0.001; *ns*, not significant (*P*>0.05); compared with the ‘ambient air’ groups. The results shown are the average of two biological repeats.

Our previous studies demonstrate that hypercapnic acidosis, similar as isocapnic acidosis, can activate GPR4 to increase the expression of adhesion molecules (E-selectin, VCAM-1 and ICAM-1) and the adhesiveness of HUVEC [13]. Here we examined whether hypercapnic acidosis could stimulate the expression of other inflammatory genes identified by the microarray analyses. As shown in Fig. 4, hypercapnic acidosis treatment of HUVEC/Vector cells induced the gene expression of CXCL2, CCL20, IL8, and CD69, and the increase of expression was further augmented by GPR4 overexpression in HUVEC/GPR4 cells, showing a similar pattern as the effects of isocapnic acidosis on HUVEC. However, there were some differences as well. For example, hypercapnic acidosis failed to increase the expression of PTGS2 in HUVEC/Vector cells; gene expression level was actually even lower upon hypercapnic acidosis (20% CO_2_) treatment compared to the ambient air treatment (Fig. 4E). But the overexpression of GPR4 could still increase PTGS2 expression upon hypercapnic acidosis in HUVEC/GPR4 cells. The reason why the expression of PTGS2 in HUVEC at alkaline pH (ambient air treatment) is higher than that at pH 7.4 (5% CO_2_) is currently unknown. The expression of IL1A was induced by hypercapnic acidosis in HUVEC/GPR4 cells but unchanged in HUVEC/Vector cells (Fig. 4D).

### Acidosis activation of GPR4 also stimulates inflammatory gene expression in human lung microvascular endothelial cells and human pulmonary artery endothelial cells

As different types of vascular endothelial cells may exhibit different biological responses [24], we examined whether activation of GPR4 by acidosis can increase the expression of inflammatory genes in other endothelial cells in addition to HUVEC. As shown by RT-PCR (Fig. S1A), GPR4 was the predominant member of the proton-sensing GPCRs expressed in HUVEC, primary human pulmonary artery endothelial cells (HPAEC) and human lung microvascular endothelial cells (HMVEC-L). Other proton-sensing GPCRs, including TDAG8 (GPR65), OGR1 (GPR68) and G2A (GPR132), were expressed at very low level in these endothelial cells. Compared to GPR4 expression in HUVEC (set as 100%), real-time RT-PCR showed that GPR4 was expressed in HPAEC and HMVEC-L at the level of∼120% and∼47%, respectively (Fig. S1B). Attempts were made to detect the protein expression of GPR4 by Western blotting, immunofluorescence, and flow cytometry using several commercially available GPR4 antibodies; however, we have not yet been able to obtain definitive GPR4-specific signals using these antibodies. This represents a limitation for the GPR4 study.

HPAEC and HMVEC-L were treated with pH 8.4 (4 nM H^+^), pH 7.4 (40 nM H^+^), or pH 6.4 (400 nM H^+^) for 5 hours, and the expression of several inflammatory genes was assessed. The results showed that, in comparison to pH 8.4 and 7.4, pH 6.4 stimulated the expression of these inflammatory genes except for ICAM-1 in HPAEC and HMVEC-L (Fig. S2). This observation is in accordance with the results in HUVEC. Interestingly, the basal expression level of the inflammatory genes was higher in HMVEC-L than that in HPAEC. However, the fold of increase in gene expression was larger in HPAEC than that in HMVEC-L (Fig. S2). This is consistent with the higher expression level of GPR4 in HPAEC (Fig. S1B).

**Figure 5:**
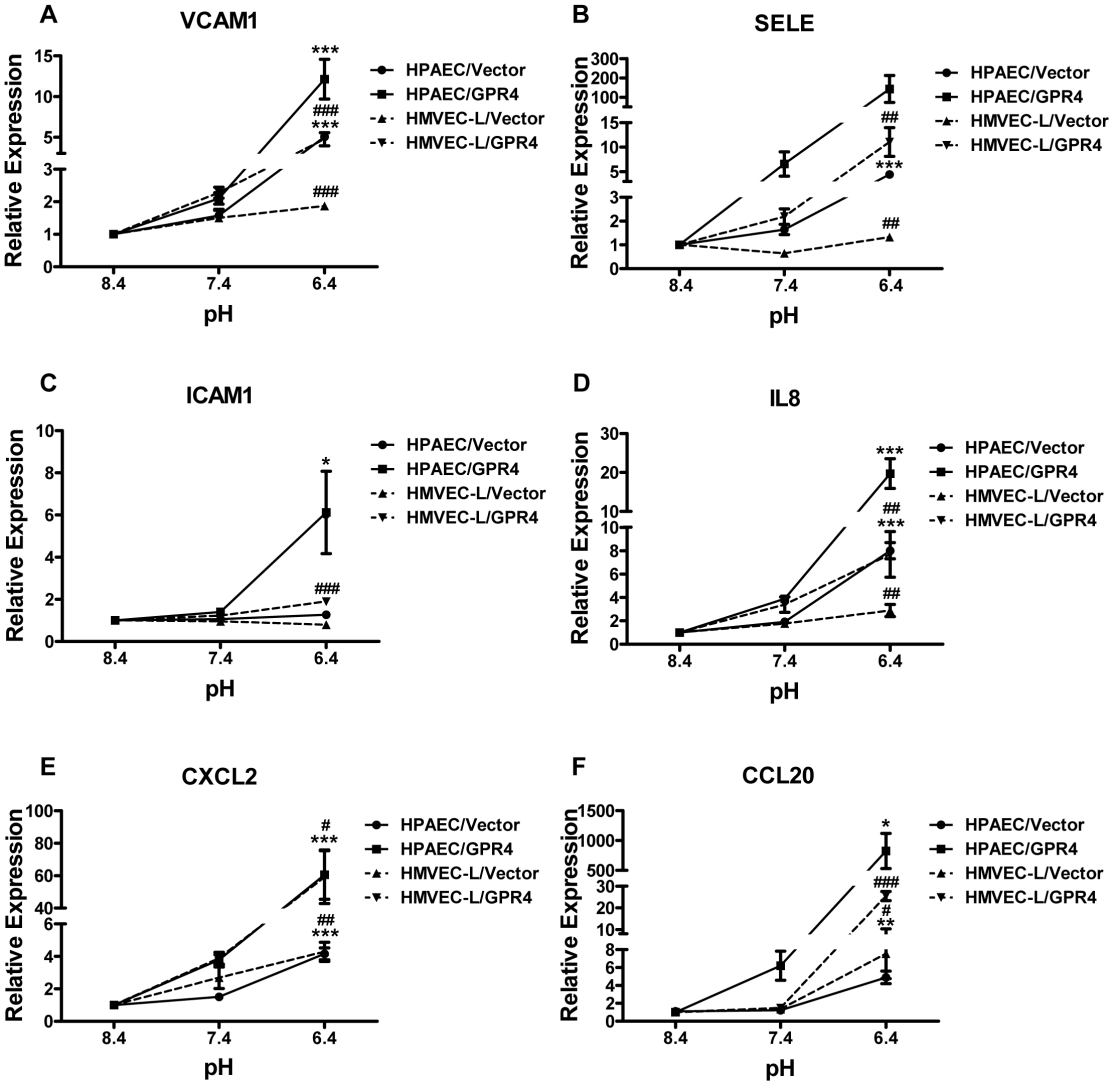
Isocapnic acidosis activation of GPR4 also increases the expression of inflammatory genes in HPAEC and HMVEC-L. HPAEC or HMVEC-L cells were transduced with the control vector or the GPR4 expression construct (designated as HPAEC/Vector (•), HPAEC/GPR4 (▪), HMVEC-L/Vector (▴), and HMVEC-L/GPR4 (▾)). HPAEC or HMVEC-L cells were then treated with EGM-2/HEM or EGM-2-MV/HEM media at pH 8.4, 7.4, or 6.4 for 5 h, respectively. Real-time RT-PCR quantification of mRNA levels of VCAM1 (A), SELE (B), ICAM1 (C), IL8 (D), CXCL2 (E) and CCL20 (F) was performed. The expression level of the target genes in above-mentioned cells at pH 8.4 was set as 1. Error bars indicate the mean ± SEM. *, *P*<0.05; **, *P*<0.01; ***, *P*<0.001; comparing pH 6.4 to pH 8.4 in HPAEC cells. #, *P*<0.05; ##, *P*<0.01; ###, *P*<0.001; comparing pH 6.4 to pH 8.4 in HMVEC-L cells. The results shown are the average of at least two biological repeats.

To investigate whether overexpression of GPR4 can further increase the expression of the inflammatory genes in response to acidosis, HPAEC and HMVEC-L were stably transduced with human GPR4 cDNA or the MSCV-IRES-GFP vector control. As shown in Fig. 5, upon acidosis treatment, HPAEC/Vector cells showed a similar extent of up-regulation of the inflammatory genes expression as HPAEC parental cells did (Fig. S2), while HPAEC/GPR4 cells, with GPR4 overexpression, showed a much stronger up-regulation of those inflammatory genes (Fig. 5). Similar results were observed in HMVEC-L/Vector and HMVEC-L/GPR4 cells (Fig. 5). Taken together, these results suggest that acidosis also stimulates GPR4 to increase the expression of inflammatory genes in human pulmonary artery endothelial cells and human lung microvascular endothelial cells.

### The NF-κB pathway is important for acidosis/GPR4-induced inflammatory gene expression in endothelial cells

**Figure 6:**
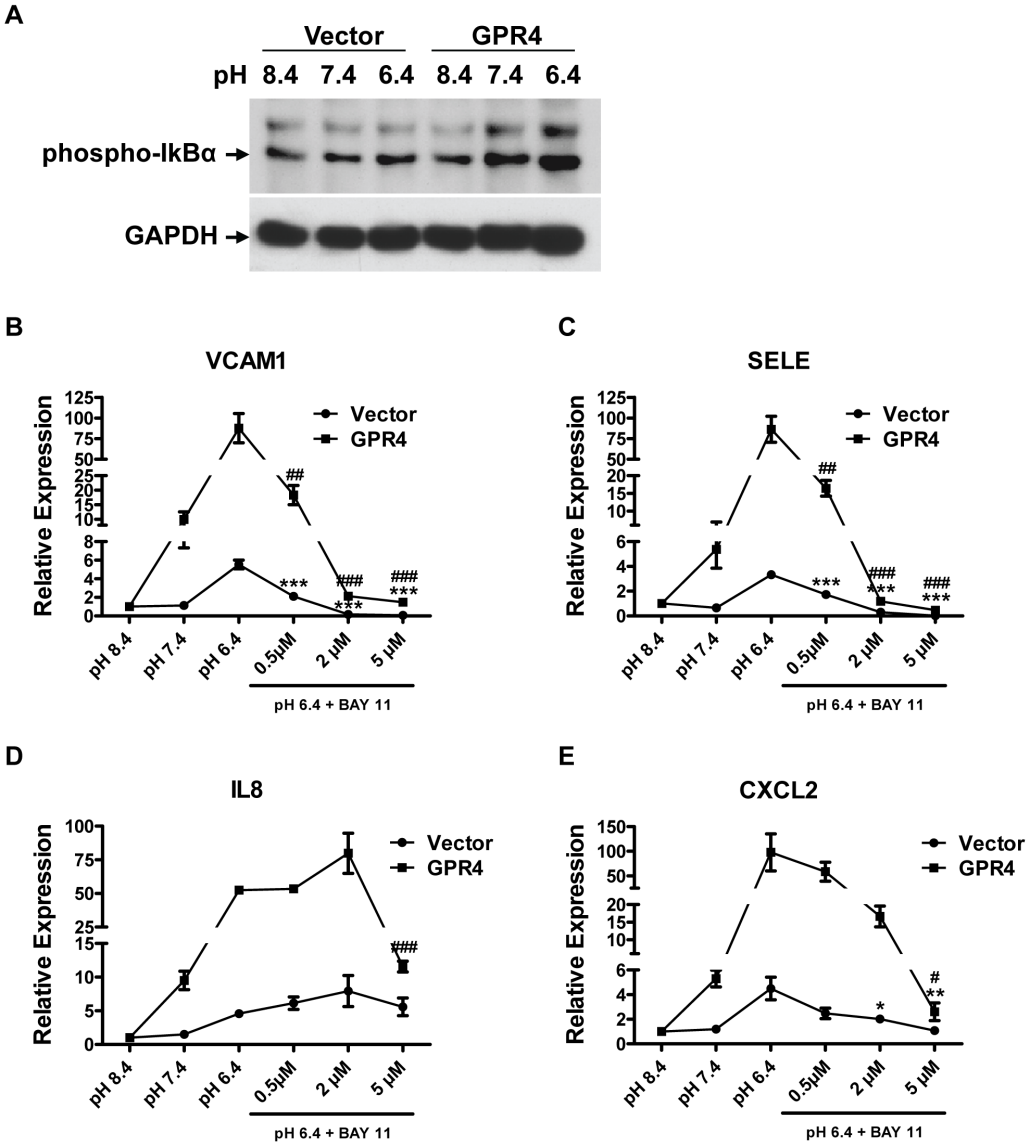
NF-κB pathway is involved in acidosis/GPR4-induced inflammatory response. (A) Western blot of phosphorylated IkB-α expression in HUVEC/Vector and HUVEC/GPR4 cells. Cells were pretreated with EGM-2/HEM pH 8.4 medium for 4 h, followed by the treatment with EGM-2/HEM media at pH 8.4, 7.4, or 6.4 for 3 min. The target bands are indicated by an arrow. Western blot of GAPDH serves as a loading control. (B–E) HUVEC/Vector or HUVEC/GPR4 cells were treated for 5 h with EGM-2/HEM pH 8.4, 7.4 or 6.4 media, or with pH 6.4 media containing indicated concentrations of NF-κB inhibitor BAY 11-7082. Total RNA was isolated and cDNA was synthesized. Real-time RT-PCR quantification of gene expression of VCAM1 (B), SELE (C), IL8 (D) and CXCL2 (E) was performed. Ct values were normalized to the housekeeping gene GAPDH. The expression level of the target genes at pH 8.4 was set as 1. Error bars indicate the mean ± SEM. *, *P*<0.05; **, *P*<0.01; ***, *P*<0.001; compared with the pH 6.4 vehicle control in HUVEC/Vector cells. #, *P*<0.05; ##, *P*<0.01; ###, *P*<0.001; compared with the pH 6.4 vehicle control in HUVEC/GPR4 cells. The results shown are the average of at least three biological repeats.

As the microarray analysis showed that several genes in the NF-κB pathway were up-regulated by acidosis activation of GPR4 in HUVECs, we investigated whether the NF-κB pathway was important for the acidosis/GPR4-induced inflammatory gene expression. Western blotting analysis showed that acidosis stimulation of GPR4 quickly increased the phosphorylation of IκB-α in HUVECs within 3 minutes (Fig. 6A), indicating the activation of the NF-κB pathway [25]. Furthermore, HUVEC/Vector and HUVEC/GPR4 cells were treated with two different NF-κB inhibitors: BAY11-7082 that inhibits IκB-α phosphorylation and the IKK inhibitor VII. Treatment with NF-κB inhibitors substantially abolished the acidosis/GPR4-induced inflammatory gene expression in HUVEC/Vector and HUVEC/GPR4 cells in a dose-dependent manner (Fig. 6 and Fig. S3), suggesting an important role for the NF-κB pathway in this process. Notably, compared to VCAM1, SELE, and CXCL2, the expression of IL8 was less sensitive to the BAY11-7082 inhibition especially in HUVEC/Vector cells (Fig. 6D), suggesting that other pathways besides NF-κB might also be important for IL8 expression [26].

### Acidosis/GPR4-induced endothelial cell inflammation enhances the binding with U937 monocytes under a flow condition

**Figure 7:**
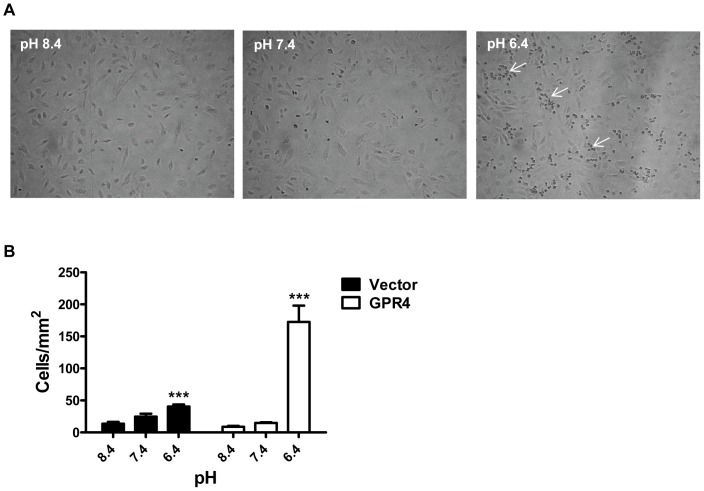
Increased binding of U937 monocytes to vascular endothelial cells treated with acidic pH. (A) U937 monocytic cells were adherent to acidic pH-treated HUVEC/GPR4 cells under a flow condition. Representative pictures are shown with the adhered U937 cells indicated by arrows. (B) HUVECs stably overexpressing GPR4 or control vector were grown to a monolayer, and were treated with EGM-2/HEM pH 8.4, 7.4 or 6.4 media for 5 h. U937 monocytes were adhered to the pH-treated HUVEC monolayer under a flow condition (0.5 dyne/cm^2^). Error bars indicate the mean ± SEM. ***, *P*<0.001; compared with the pH 8.4 groups. The results represent the average of cell counts from 6 fields.

The microarray and real-time PCR results showed that activation of GPR4 by acidosis stimulated the expression of a number of inflammatory adhesion molecules and chemokines (Table 1). Therefore, we examined whether acidosis/GPR4-induced endothelial cell inflammation would increase the binding with U937 monocytes under a flow condition at the wall shear stress of 0.5 dyne/cm^2^. For HUVECs treated with pH 8.4 and pH 7.4, there was little firm adhesion or rolling of U937 monocytes under the flow condition. In comparison, U937 monocytes showed increased rolling and adherence to pH 6.4-treated HUVEC/Vector cells and this effect was substantially further enhanced in pH 6.4-treated HUVEC/GPR4 cells (Fig. 7 and Videos S1–S6). These observations from the flow chamber assay were concordant with our previous static cell adhesion findings [13]. Together, the results show that acidosis activation of GPR4 in HUVECs augments the binding to U937 monocytes.

### Inhibition of GPR4 by its antagonist attenuates acidosis-induced endothelial cell inflammation

**Figure 8:**
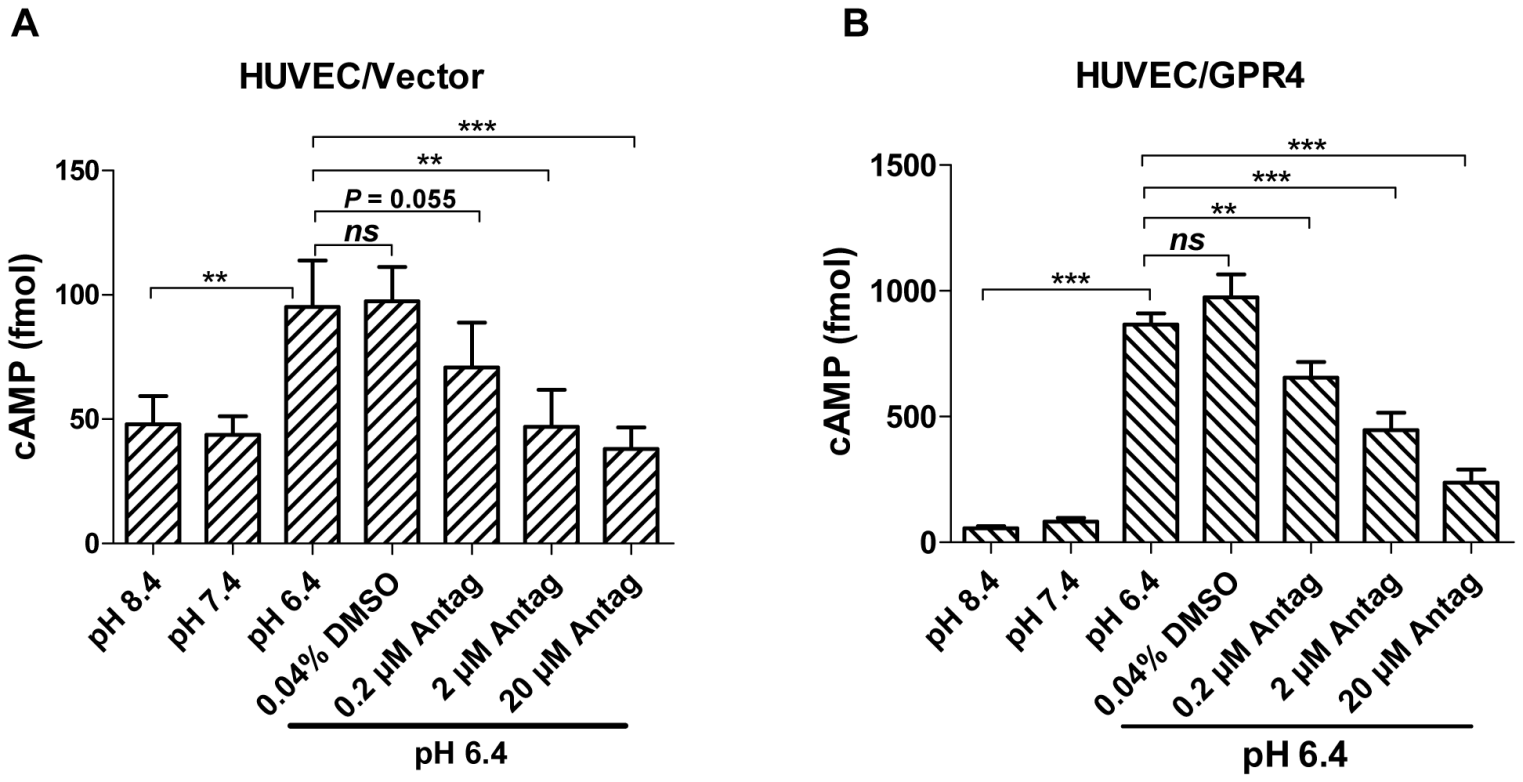
Inhibition of acidosis/GPR4-induced cAMP production by the GPR4 antagonist in HUVEC. (A–B) HUVEC/Vector and HUVEC/GPR4 cells were treated with varying pH in the presence or absence of the GPR4 antagonist EIDIP. After the pH treatment, intracellular cAMP was measured as described in the Materials and Methods. The vehicle control had 0.04% DMSO which is the same DMSO concentration as that in 20 µM GPR4 antagonist. The results are the average of 10 samples for HUVEC/Vector cells and 7 samples for HUVEC/GPR4 cells. Error bars indicate the mean ± SEM. **, *P*<0.01; ***, *P*<0.001; *ns*, not significant.

**Figure 9:**
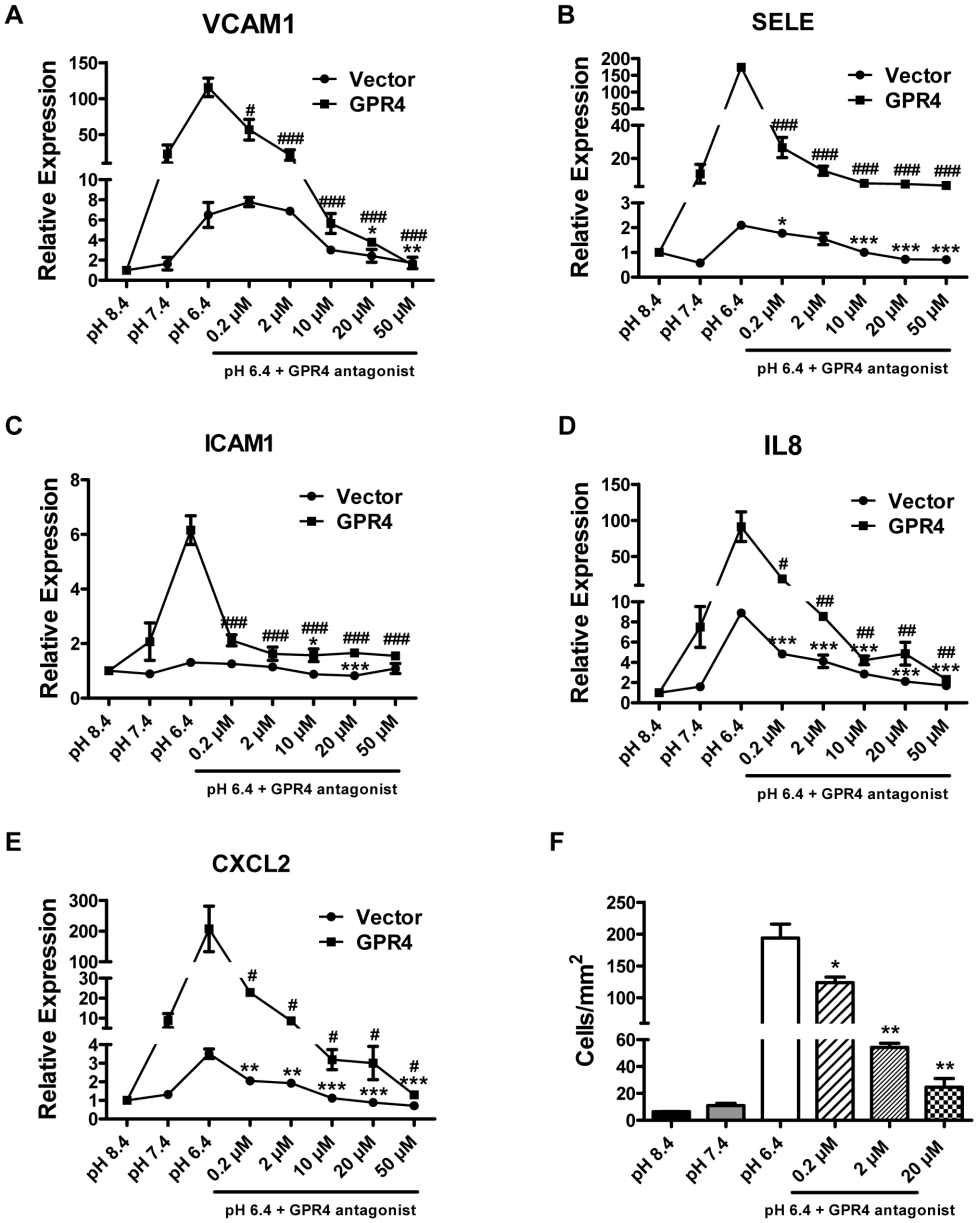
Inhibition of GPR4 activation by its antagonist attenuates the expression of inflammatory genes. (A–E) HUVEC/Vector or HUVEC/GPR4 cells were treated for 5 h with EGM-2/HEM pH 8.4, 7.4 or 6.4 media, or with pH 6.4 media containing indicated concentrations of GPR4 antagonist. Total RNA was isolated and cDNA was synthesized. Real-time RT-PCR quantification of gene expression of VCAM1 (A), SELE (B), ICAM1 (C), IL8 (D) and CXCL2 (E) was performed. The expression level of the target gene in HUVECs at pH 8.4 was set as 1. Error bars indicate the mean ± SEM. *, *P*<0.05; **, *P*<0.01; ***, *P*<0.001; compared with the pH 6.4 vehicle control in HUVEC/Vector cells. #, *P*<0.05; ##, *P*<0.01; ###, *P*<0.001; compared with the pH 6.4 vehicle control in HUVEC/GPR4 cells. The results shown are the average of at least two biological repeats. (F) HUVECs stably overexpressing GPR4 were grown to form a monolayer. Cells were then pretreated with vehicle or GPR4 antagonist (at indicated concentrations) for 1 h, followed by the treatment with indicated pH media or pH 6.4 medium containing indicated concentrations of GPR4 antagonist for 5 h. The static cell adhesion assay was then performed using U937 monocyte binding as a functional readout as previously described [13]. Error bars indicate the mean ± SEM. *, *P*<0.05; **, *P*<0.01; compared with the pH 6.4 vehicle group. The results represent the average of cell counts from 3 fields under an inverted microscope (total 100× magnification).

A group of imidazo-pyridine derivatives were recently identified as GPR4 antagonists that inhibit GPR4 activities [27]. However, the effects of the GPR4 inhibitors on endothelial cell inflammation have not been examined. We assessed the biological effects of one of the antagonist compounds, 2-Ethyl-3-{4-[(E)-3-(4-isopropyl-piperazin-1-yl)-propenyl]-benzyl}-5,7-dimethyl-3H-imidazo[4,5-b]pyridine (abbreviated as EIDIP), in our study. We first examined whether EIDIP can inhibit the acidosis/GPR4-mediated cAMP production in endothelial cells. HUVEC/Vector and HUVEC/GPR4 cells were treated with varying pH in the presence or absence of the GPR4 antagonist EIDIP. Compared to pH 8.4 and 7.4, pH 6.4 increased intracellular cAMP accumulation by∼2 fold and∼10 fold in HUVEC/Vector and HUVEC/GPR4 cells, respectively. The treatment with EIDIP significantly inhibited the acidosis/GPR4-induced cAMP accumulation (Fig. 8). Furthermore, we showed that the treatment of the GPR4 inhibitor diminished the acidosis/GPR4-induced inflammatory gene expression in HUVEC/Vector and HUVEC/GPR4 cells in a dose-dependent manner (Fig. 9A–9E). The GPR4 inhibitor treatment also decreased the acidosis/GPR4-induced HUVEC adhesiveness as measured by the U937 cell adhesion assay (Fig. 9F). These results suggest that inhibition of GPR4 by its antagonist can attenuate acidosis-stimulated endothelial cell inflammation.

## Discussion

The major findings of this study are that acidosis activation of the proton-sensing receptor GPR4 stimulates a broad inflammatory response in human vascular endothelial cells and the treatment with a recently identified inhibitor of GPR4 can effectively suppress this inflammatory response. These results indicate that the acidosis/GPR4 receptor signaling is a novel pathway in endothelial cell inflammatory response, which is a crucial component in many pathological conditions such as inflammation, ischemia, sickle cell disease, tumor, metabolic diseases, renal diseases, and respiratory diseases [1], [2], [3], [4], [28]. This aspect of biology is very relevant because local or systemic acidosis is a hallmark of tissue microenvironment in these diseases [6], [8], [10], [12], [29].

Numerous studies have shown that tissue acidity aggravates organ injury and exacerbates the progression of acidosis-associated diseases such as ischemic diseases and sickle cell disease [8], [10], [30]. In ischemic heart disease and stroke, inflammation plays a pivotal role in promoting tissue damage after ischemia and reperfusion. Leukocyte infiltration is commonly observed in ischemic tissues, and reactive oxygen species and proteolytic enzymes produced by leukocytes are important mediators of tissue damage [31], [32]. Leukocyte adhesion to the microvasculature is rapidly enhanced during the reperfusion process [33]. Leukocyte and platelet intravascular plugging may play a role in the “no reflow” phenomenon following blood reperfusion [34]. Based on our results, it is tempting to speculate that acidosis/GPR4-induced endothelial cell adhesion and inflammatory response may increase the recruitment and activation of leukocytes in ischemic tissues and may also contribute to vaso-occlusion and the “no reflow” phenomenon after reperfusion. As another example, acidosis is a well-known risk factor that increases the incidence of sickle cell crisis and vaso-occlusive events in sickle cell disease [30], [35], [36], [37]. Previous studies show that acidosis increases red cell sickling and dense sickle erythrocyte formation [30], [35]. However, this may not be the only mechanism responsible for vaso-occlusion. In fact, our results suggest a potential novel mechanism by which acidosis aggravates sickle cell crisis; that is, acidosis activates the proton-sensing GPR4 receptor to stimulate endothelial cell inflammatory response and thus increases blood cell attachment and vaso-occlusion. Concordant with this thought, endothelial cell inflammation is correlated with an increased risk of stroke and occlusive disease at the circle of Willis in sickle cell patients [38].

Acidosis activation of the GPR4 receptor in vascular endothelial cells modulates the expression of a large number of genes involved in various biological pathways, such as inflammation, endoplasmic reticulum (ER) stress response, and cell metabolism. In line with our observations, a recent study shows that lactic acidosis induces inflammatory response and ER stress response in breast cancer cells [9], although many of the up-regulated target genes are different between endothelial cells and breast cancer cells. Our results provide evidence that the proton-sensing GPCRs are involved in acidosis-induced ER stress response, a process closely related to inflammation [39]. Moreover, two earlier studies show that chronic acidosis treatment regulates the expression of genes involved in cell metabolism in renal cells and intestinal cells [40], [41]. All these studies suggest some common features of acidosis responses in various cell types. On the other hand, certain acidosis responses are particularly prominent in a cell type-specific manner. For instance, the up-regulation of inflammatory chemokines, cytokines and adhesion molecules are particularly strong in vascular endothelial cells that we have studied. Our results also indicate that the proton-sensing GPCRs, such as GPR4, can at least partly mediate the acidosis-induced cell responses.

Acidosis/GPR4-mediated inflammatory gene expression appears to be general in several types of vascular endothelial cells. In addition to HUVEC, human lung microvascular endothelial cells and human pulmonary artery endothelial cells can also be stimulated by acidosis to up-regulate inflammatory molecules. Genetic overexpression of GPR4 in these endothelial cells substantially further increases the acidosis-induced inflammatory gene expression. These results show that the activation of GPR4 by acidosis triggers a pro-inflammatory signal cascade in endothelial cells. In the literature, the effects of acidosis on vascular endothelial cells remain largely unclear and, in some cases, opposite effects have been reported. For instance, a previous study shows that hypercapnic acidosis decreases the lipopolysaccharide-induced ICAM-1 expression in human pulmonary artery endothelial cells [42]. On the other hand, a more recent study observes the opposite effects and shows that hypercapnic acidosis increases the lipopolysaccharide-induced expression of ICAM-1, VCAM-1 and E-selectin in human lung microvascular endothelial cells [43]. In our current study, the unbiased genome-wide microarray analysis clearly demonstrates that acidosis activation of GPR4 in endothelial cells induces the expression of a broad range of inflammatory genes including adhesion molecules, chemokines and cytokines. For ICAM-1 in particular, acidic pH did not significantly affect its expression in the vector control endothelial cells but still substantially increased the expression of ICAM-1 in GPR4-overexpressing cells, indicating that ICAM-1 is also an acidosis/GPR4-induced gene. Overall, our data strongly suggest that acidosis activation of GPR4 stimulates a pro-inflammatory response in vascular endothelial cells.

As the interaction between endothelial cells and blood cells is critical for inflammation and vaso-occlusion, targeting endothelial cell inflammation has been exploited as a strategy for the treatment of inflammatory diseases and vaso-occlusive diseases. For example, antibodies and small molecules targeting endothelial cell adhesion molecules have been tested for the treatment of inflammation, sickle cell disease, stroke and ischemic heart disease [2], [3], [4], [44]. GPR4 may serve as a novel target for the inhibition of endothelial cell inflammatory response. In this respect, our results show that a recently identified GPR4 inhibitor can suppress the acidosis-induced inflammatory gene expression in endothelial cells. We have also previously shown that inhibition of GPR4 by small interfering RNAs attenuates acidosis-induced endothelial cell adhesion [13]. Furthermore, our results suggest that the NF-κB pathway is important for the acidosis/GPR4-induced inflammatory gene expression in endothelial cells. In complex diseases such as chronic inflammation and sickle cell disease, many pathogenic factors are involved. Effective, chronic medication with minimal side effect is highly desirable for the management of this type of disease. With this regard, GPR4 represents a potential therapeutic target whose inhibition may have acceptable safety profile since the phenotype of GPR4-deficient mice is mild compared to the knockout phenotype of some critical regulators of inflammation [45], [46]. However, it should be noted that GPR4-null mice have minor defects in renal acid excretion and mild metabolic acidosis [47]. A small fraction of GPR4-null mice exhibit a partially penetrant phenotype of perinatal mortality in a mixed B6/129 genetic background [15]. GPR4 deficiency also affects the quality of small blood vessels during angiogenesis [14], [15]. Nonetheless, future research is warranted to validate GPR4 as a potential therapeutic target for inhibiting inflammation and vaso-occlusion in acidosis-associated diseases. Plausibly, combination therapy that targets multiple molecular pathways with acceptable drug safety profile is needed to treat complex diseases such as inflammatory and vaso-occlusive disorders. GPR4 inhibitors may be exploited as potential novel agents to suppress vascular inflammatory responses.

## Materials and Methods

### Chemicals and reagents

Real-time PCR reagents were purchased from Applied Biosystems Inc (ABI, Foster City, CA). 4-(2-hydroxyethyl)-1-piperazineethanesulfonic acid (HEPES), *N*-(2-hydroxyethyl)-piperazine-*N'*-3-propanesulfonic acid (EPPS), 2-(4-morpholino)-ethanesulfonic acid (MES), and protease inhibitor cocktail were from Sigma-Aldrich (St Louis, MO) and Fisher Scientific (Fair Lawn, NJ). BAY 11-7082 and IKK inhibitor VII were purchased from Calbiochem/EMD4Biosciences (La Jolla, CA). GPR4 antagonist, 2-Ethyl-3-{4-[(E)-3-(4-isopropyl-piperazin-1-yl)-propenyl]-benzyl}-5,7-dimethyl-3H-imidazo[4,5-b]pyridine, was purchased from Dalton Pharma Services (Toronto, Canada). The Amersham cAMP Biotrak Enzymeimmunoassay (EIA) kit was purchased from GE Healthcare Life Sciences. 0.1% gelatin in ultrapure water was from Millipore (Billerica, MA). Monoclonal antibodies for phosphorylated IκB-α (Ser32) (clone 14D4) and GAPDH (clone 14C10) were from Cell Signaling Technology (Danvers, MA). Polyclonal antibodies for DDIT3 (a.k.a. GADD 153, CHOP) and PTGS2 (COX-2) were from Santa Cruz Biotechnology (Dallas, TX).

### Cell culture and retroviral transduction

Cells were cultured in a humidified tissue culture incubator filled with 5% CO_2_ and 95% air at 37 °C. Primary human umbilical vein endothelial cells (HUVEC), human lung microvascular endothelial cells (HMVEC-L) and human pulmonary artery endothelial cells (HPAEC) were purchased from Lonza (Walkersville, MD). HUVECs and HPAECs were grown in endothelial cell growth medium 2 (EGM-2), and HMVEC-Ls were grown in EGM-2-MV medium (Lonza). The construction of the MSCV-huGPR4-IRES-GFP plasmid and the retroviral transduction of HUVEC, HPAEC or HMVEC-L cells were performed as previously described [5], [13]. Human endothelial cells stably expressing the MSCV-IRES-GFP or MSCV-huGPR4-IRES-GFP construct were isolated by fluorescence-activated cell sorting (FACS) based on green fluorescence signals.

### Isocapnic and hypercapnic pH treatment

The preparation of isocapnic pH media was carried out as previously described [13]. Briefly, EGM-2 or EGM-2-MV media were buffered with 7.5 mM HEPES, 7.5 mM EPPS and 7.5 mM MES (abbreviated as HEM), and the pH was adjusted using NaOH or HCl and measured with an electronic pH meter (Fisher). To prepare hypercapnic pH media, regular EGM-2 medium was added in cell culture plates and incubated overnight in humidified tissue culture incubators with ambient air, 5% CO_2_ or 20% CO_2_, respectively. The pH of the media pre-treated under these conditions was measured to be around 8.4, 7.4 and 6.4, respectively. Before the pH treatment, human endothelial cells were cultured in 10-cm plates, 6-cm plates or 6-well plates to reach 50–90% confluency. To perform isocapnic pH treatment, endothelial cells were incubated for 5–6 hours in the EGM-2/HEM, or EGM-2-MV/HEM media at varying pH in a regular tissue culture incubator with 5% CO_2_. To perform hypercapnic pH treatment, endothelial cells were treated with CO_2_-buffered EGM-2 media for 5–6 hours in tissue culture incubators with 20% CO_2_ and with ambient air and 5% CO_2_ as controls. When an inhibitor or antagonist was used, cells were pretreated with regular growth medium containing indicated concentrations of inhibitor or antagonist for 1 hour, followed by 5 hours of pH treatment with HEM-buffered growth medium containing same concentrations of inhibitor or antagonist.

### Microarray hybridization and analysis

Microarray was performed on Agilent 4×44K human whole genome microarray chips at the Genomics and Bioinformatics Core of the University of North Carolina, Chapel Hill. Human 4×44K whole genome microarray chips were purchased from Agilent Technologies (Santa Clara, CA). HUVEC/Vector cells and HUVEC/GPR4 cells were treated with pH 6.4 for 5 hours to activate GPR4 or with pH 8.4 for 5 hours as a negative control. After the pH treatment, total RNA was isolated using the RNeasy Plus Mini Kit (QIAGEN, Valencia, CA) following the manufacturer's protocol. RNA quality was assessed by electrophoresis using the Agilent 2100 Bioanalyzer and the RNA samples of high integrity number were used for microarray hybridization. Total RNA from pH 6.4-treated HUVEC (vector or GPR4-overexpressing) cells was reverse transcribed and labeled with Cy5 fluorescent dye to serve as the test sample, whereas total RNA from pH 8.4-treated HUVEC (vector or GPR4-overexpressing) cells was reverse transcribed and labeled with Cy3 dye to serve as the reference control. The same amount of labeled sample and control cRNAs was hybridized to the 4×44K Agilent Whole Genome Microarray Chips. Hybridization signals were scanned using an Agilent scanner. Microarray data were normalized through LOWESS normalization and the fold of gene expression change (log_2_ ratio of the mean red intensity over mean green intensity) was analyzed using the software at the UNC Genomics and Bioinformatics Core (https://genome.unc.edu/). Significance analysis of microarrays (SAM) was used to identify genes whose expression levels were significantly altered between pH 6.4 and pH 8.4 treatments. The microarray data has been deposited to the Gene Expression Omnibus (GEO) repository under the accession number GSE40060.

### Real-time RT-PCR

Human endothelial cells with endogenous or overexpressed GPR4 levels were treated with indicated conditions. Total RNA was extracted from these cells using the RNeasy Plus mini kit (QIAGEN) and was reverse transcribed using the SuperScript II reverse transcriptase (Invitrogen, CA). TaqMan pre-designed primer-probes specific for target genes (Applied Biosystems) were listed in the Table S1. The primer-probes for human GPR4 and glyceraldehydes-3-phophate dehydrogenase (GAPDH) have been previously described [13]. Real-time PCR was performed in duplicate with a program of 50°C for 2 min, 95°C for 10 min followed by 40 cycles of 95°C for 15 sec and 60°C for 1 min, and the data was acquired and analyzed using the ABI 7300 or ABI 7900HT real-time PCR thermocycler. The fold of gene expression changes was calculated using the 2^−ΔΔCt^ method [22].

### Western blotting

After indicated length of pH treatment, endothelial cells were lysed in ice-cold radioimmune precipitation assay (RIPA) buffer as previously described [13]. Protein concentration of cell lysate supernatant was determined by the Bradford protein assay (Bio-Rad, Hercules, CA) or the bicinchoninic acid (BCA) assay kit (Thermo Scientific). Cell lysates were then separated by SDS-PAGE and transferred onto nitrocellulose membrane (GE Healthcare). The expression of phosphorylated IκB-α, DDIT3 (CHOP), PTGS2 (COX-2) and GAPDH was analyzed by Western blotting with corresponding primary antibodies and the horseradish peroxidase (HRP)-conjugated secondary antibody (Santa Cruz Biotechnology). Chemiluminescence signals were detected using the Amersham ECL Advance Western blotting detection kit according to the manufacturer's instruction (GE Healthcare).

### Intracellular cAMP measurement

The cAMP assay was performed as previously described [13]. Briefly, HUVEC/Vector or HUVEC/GPR4 cells were seeded at a 2.5×10^4^ cells/well (100 µl) in a 96-well plate and allowed to attach overnight. The following day old media were removed and cells were pretreated for 10 minutes at 37°C in a tissue culture incubator with the following conditions: EGM-2 or EGM-2 with 0.04% DMSO, 0.2 µM GPR4 antagonist, 2 µM GPR4 antagonist, or 20 µM GPR4 antagonist in a total volume of 100 µl per well. After the pretreatment, media were removed and cells were treated for 10 minutes at 37°C in a tissue culture incubator with the following conditions: EGM-2/HEM buffered to pH 8.4, pH 7.4, and pH 6.4, pH 6.4+0.04% DMSO, pH 6.4+0.2 µM GPR4 antagonist, pH 6.4+2 µM GPR4 antagonist, and pH 6.4+20 µM GPR4 antagonist. All treatment media contained 0.5 mM IBMX. After the pH treatment, intracellular cAMP was measured using the Amersham cAMP Biotrak Enzymeimmunoassay (EIA) System (GE Healthcare Life Sciences Cat # RPN2251) following the protocol provided by the manufacturer.

### Flow chamber cell adhesion assay

HUVEC/Vector or HUVEC/GPR4 cells were cultured on 6-cm plates that were pre-coated with 0.1% gelatin to form a monolayer. Growth medium was then switched to EGM-2/HEM media at pH 8.4, 7.4 or 6.4, and HUVECs were treated for 5 hours in the pH medium. A parallel plate flow chamber kit (GlycoTech, Gaithersburg, MD) was used to perform dynamic flow chamber assays according to the manufacturer's protocol. The plastic chamber was placed on to a rubber gasket which has the thickness of 0.01 inches and a flow path width of 0.5 cm. These dimensions allow for the cells to be flowed through the chamber with a wall shear stress of 0.5 dyne/cm^2^ at a volumetric flow rate of 0.2 ml/min. The flow chamber was connected to a programmable syringe pump (Harvard Apparatus). Once the chamber was set up, one plate of the treated HUVECs was washed once with EGM-2, and then media were added on top of the monolayer of HUVECs to keep them viable during the assay. U937 monocytic cells (ATCC, Manassas, VA) at the concentration of 1×10^6^ cells/ml were then flowed through the chamber for 5 minutes while video was taken. After 5 minutes of flowing, the syringe containing the cells was removed and replaced with a syringe containing warm RPMI+10% FBS. Cells were then washed for another 3 to 5 minutes until all non-adhered cells were removed. After the wash, still images of 3 fields of adherent U937 cells were taken for each treatment condition. Images were then analyzed using Adobe Photoshop and adherent U937 cells were counted (total 22.4×magnification, approximately area of 1.2 mm^2^ per image). Static HUVEC-U937 cell adhesion was performed as previously described [13], and attached U937 cells from 3 fields were counted under an inverted microscope (total 100× magnification, approximately area of 2.7 mm^2^ per 100× field).

### Statistical analysis

Data was analyzed using the GraphPad Prism 5 software. The results of cAMP production, cell adhesion, and a substantial part of real-time RT-PCR data points were derived from three or more independent biological replicates, whereas some real-time RT-PCR results were based on two independent biological replicates with the same trend of gene expression and might have a limited statistical power. *P*<0.05 (*t* test) was considered statistically significant.

## Supporting Information

Figure S1 **HUVEC, HPAEC and HMVEC-L cells have high expression level of GPR4.** (A) Total RNA was isolated from HUVEC, HPAEC or HMVEC-L parental cells, and cDNA was synthesized. Gene expression of GPR4 family members in those endothelial cells was examined by RT-PCR using gene-specific primers. (B) Total RNA was isolated from HUVEC, HPAEC or HMVEC-L parental cells, and cDNA was synthesized. Gene expression of GPR4 in those cells was examined by real-time RT-PCR. Ct values were normalized to the housekeeping gene GAPDH. The expression level of GPR4 in HUVEC was set as 1. Error bars indicate the mean ± SEM. The expression data are representative of two independent experiments.
(TIF)

Figure S2 **Isocapnic acidosis increases the expression of inflammatory genes in HPAEC and HMVEC-L.** HPAEC (white bars) or HMVEC-L (dark bars) parental cells were treated with EGM-2/HEM or EGM-2-MV/HEM media at pH 8.4, 7.4, or 6.4 for 5 h, respectively. Total RNA was isolated and cDNA was synthesized. Real-time RT-PCR quantification of gene expression of VCAM1 (A), SELE (B), ICAM1 (C), IL8 (D), CXCL2 (E) and CCL20 (F) was performed in duplicate. Ct values were normalized to the ones of housekeeping gene GAPDH. The expression level of the target gene in HPAECs at pH 8.4 was set as 1. The results are representative of two independent experiments. Error bars indicate the mean ± SEM. *, *P*<0.05; **, *P*<0.01; ***, *P*<0.001; *ns*, not significant (*P*>0.05); compared with the pH 8.4 groups.
(TIF)

Figure S3 **Inhibition of NF-κB pathway attenuates the expression of inflammatory genes.** HUVECs stably overexpressing GPR4 were treated for 5 h with EGM-2/HEM pH 8.4, 7.4 or 6.4 media, or with pH 6.4 media containing indicated concentrations of IKK inhibitor VII. Real-time RT-PCR quantification of gene expression of VCAM1 (A), SELE (B), ICAM1 (C), IL8 (D), CXCL2 (E) and CCL20 (F) was performed in duplicate. Ct values were normalized to the housekeeping gene GAPDH. The expression level of the target gene in HUVEC/GPR4 cells at pH 8.4 was set as 1. Error bars indicate the mean ± SEM. *, *P*<0.05; **, *P*<0.01; ***, *P*<0.001; *ns*, not significant (*P*>0.05); compared with the pH 6.4 vehicle groups. The results are representative of two independent experiments.
(TIF)

Table S1 **A list of TaqMan pre-designed primer-probes used in the study.**
(DOC)

Table S2 **A list of 1208 differentially expressed genes regulated by acidosis/GPR4 (values are expressed as log_2_).**
(XLS)

Table S3 **SAM analysis of genes that were induced by GPR4 overexpression in HUVECs.**
(XLSX)

Table S4 **SAM analysis of genes that were repressed by GPR4 overexpression in HUVECs.**
(XLSX)

Table S5 **Table of Gene Ontology enrichment generated by GATHER using the GPR4 overexpression-induced genes from Table S3.**
(XLSX)

Table S6 **Table of Gene Ontology enrichment generated by GATHER using the GPR4 overexpression-repressed genes from Table S4.**
(XLSX)

Table S7 **Fold changes of gene expression by real-time RT-PCR in HUVEC/Vector and HUVEC/GPR4 cells upon varying pH treatment.**
(DOC)

Video S1 **Adhesion of U937 monocytes to HUVEC/Vector cells that were treated with pH 8.4.** Adhesion of U937 monocytes to pH-treated HUVECs under a flow condition was performed as described in the “Materials and Methods”. A 15-second video clip of the HUVEC-U937 cell adhesion is presented.
(AVI)

Video S2 **Adhesion of U937 monocytes to HUVEC/Vector cells that were treated with pH 7.4.**
(AVI)

Video S3 **Adhesion of U937 monocytes to HUVEC/Vector cells that were treated with pH 6.4.**
(AVI)

Video S4 **Adhesion of U937 monocytes to HUVEC/GPR4 cells that were treated with pH 8.4.**
(AVI)

Video S5 **Adhesion of U937 monocytes to HUVEC/GPR4 cells that were treated with pH 7.4.**
(AVI)

Video S6 **Adhesion of U937 monocytes to HUVEC/GPR4 cells that were treated with pH 6.4.**
(AVI)
