# Supplementary figures and images for: Acidosis Activation of the Proton-Sensing GPR4 Receptor Stimulates Vascular Endothelial Cell Inflammatory Responses Revealed by Transcriptome Analysis
Source: PLoS One. 2013 Apr 16;8(4):e61991. doi: 10.1371/journal.pone.0061991 (PMC3628782; doi:10.1371/journal.pone.0061991)

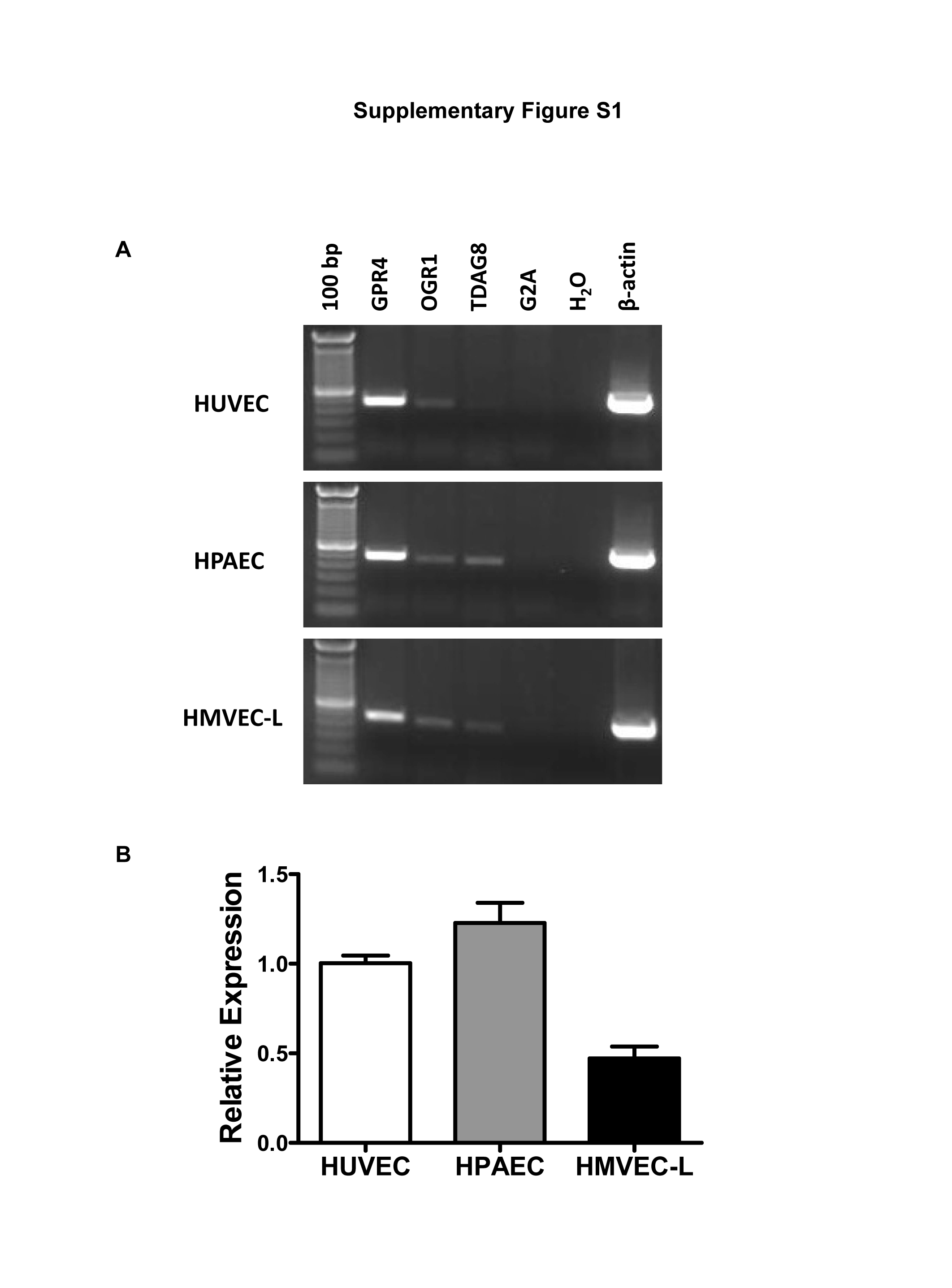

Supplement: Figure S1 — HUVEC, HPAEC and HMVEC-L cells have high expression level of GPR4. (A) Total RNA was isolated from HUVEC, HPAEC or HMVEC-L parental cells, and cDNA was synthesized. Gene expression of GPR4 family members in those endothelial cells was examined by RT-PCR using gene-specific primers. (B) Total RNA was isolated from HUVEC, HPAEC or HMVEC-L parental cells, and cDNA was synthesized. Gene expression of GPR4 in those cells was examined by real-time RT-PCR. Ct values were normalized to the housekeeping gene GAPDH. The expression level of GPR4 in HUVEC was set as 1. Error bars indicate the mean ± SEM. The expression data are representative of two independent experiments. (TIF) [file pone.0061991.s001.tif]

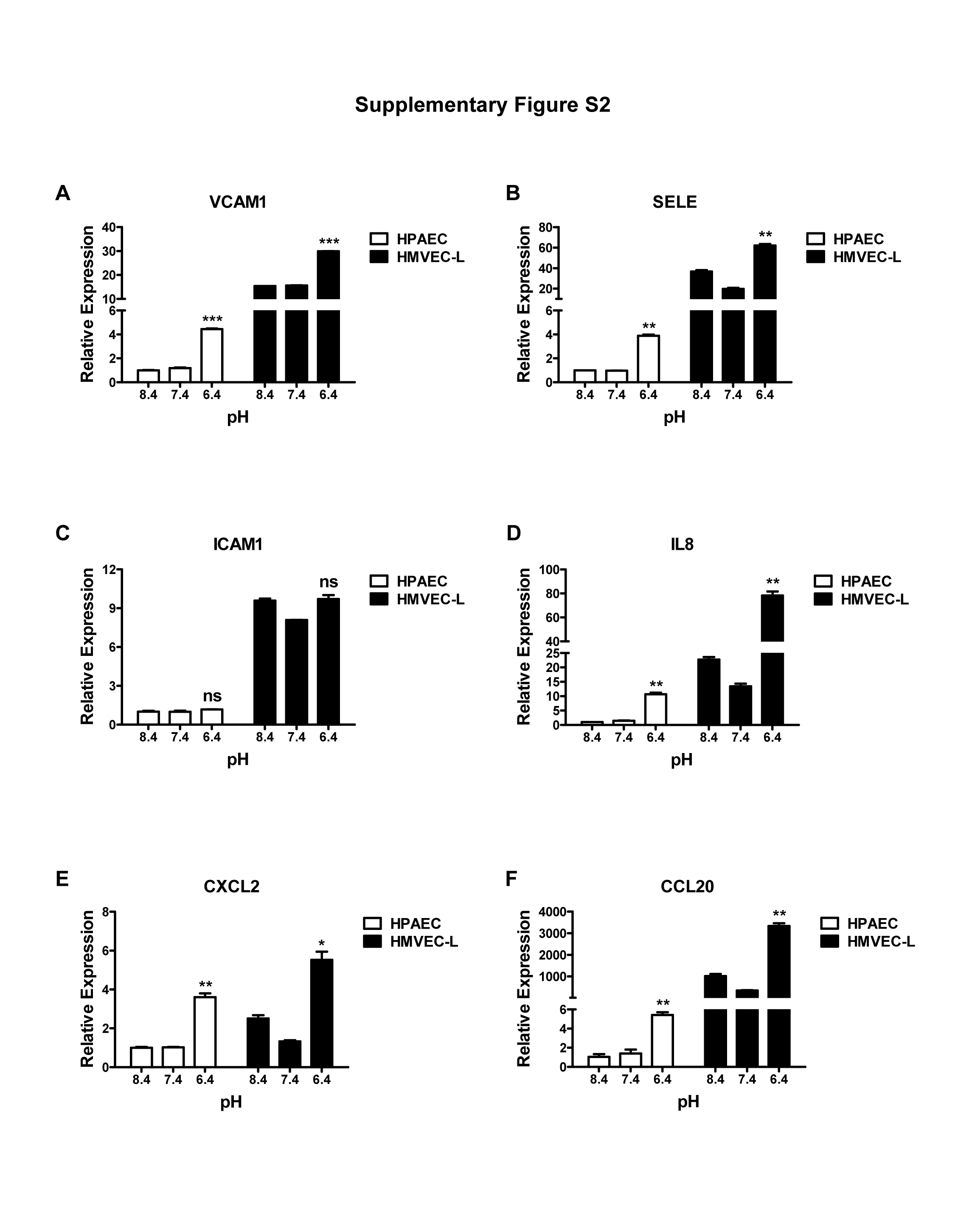

Supplement: Figure S2 — Isocapnic acidosis increases the expression of inflammatory genes in HPAEC and HMVEC-L. HPAEC (white bars) or HMVEC-L (dark bars) parental cells were treated with EGM-2/HEM or EGM-2-MV/HEM media at pH 8.4, 7.4, or 6.4 for 5 h, respectively. Total RNA was isolated and cDNA was synthesized. Real-time RT-PCR quantification of gene expression of VCAM1 (A), SELE (B), ICAM1 (C), IL8 (D), CXCL2 (E) and CCL20 (F) was performed in duplicate. Ct values were normalized to the ones of housekeeping gene GAPDH. The expression level of the target gene in HPAECs at pH 8.4 was set as 1. The results are representative of two independent experiments. Error bars indicate the mean ± SEM. *, P<0.05; **, P<0.01; ***, P<0.001; ns, not significant (P>0.05); compared with the pH 8.4 groups. (TIF) [file pone.0061991.s002.tif]

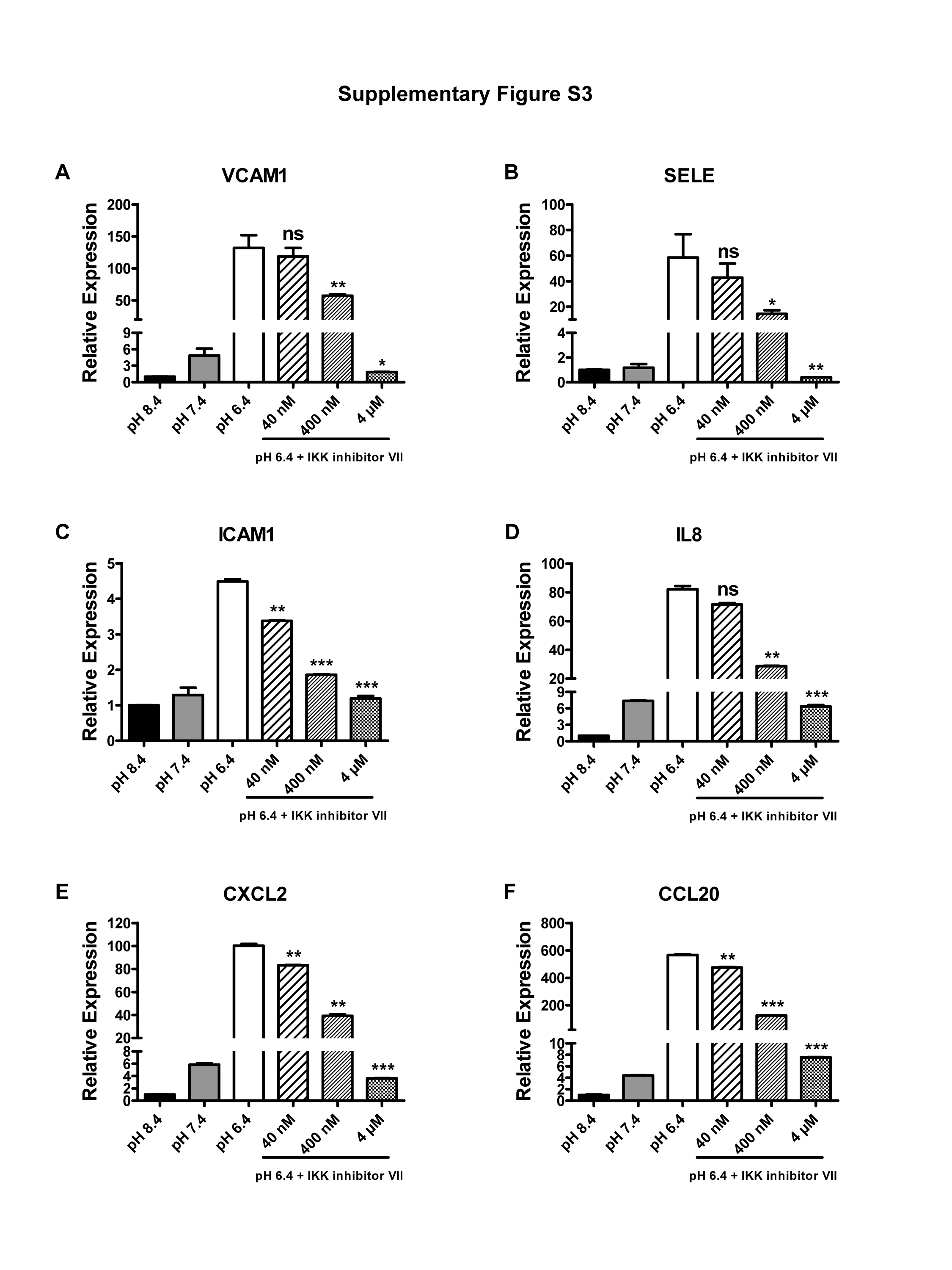

Supplement: Figure S3 — Inhibition of NF-κB pathway attenuates the expression of inflammatory genes. HUVECs stably overexpressing GPR4 were treated for 5 h with EGM-2/HEM pH 8.4, 7.4 or 6.4 media, or with pH 6.4 media containing indicated concentrations of IKK inhibitor VII. Real-time RT-PCR quantification of gene expression of VCAM1 (A), SELE (B), ICAM1 (C), IL8 (D), CXCL2 (E) and CCL20 (F) was performed in duplicate. Ct values were normalized to the housekeeping gene GAPDH. The expression level of the target gene in HUVEC/GPR4 cells at pH 8.4 was set as 1. Error bars indicate the mean ± SEM. *, P<0.05; **, P<0.01; ***, P<0.001; ns, not significant (P>0.05); compared with the pH 6.4 vehicle groups. The results are representative of two independent experiments. (TIF) [file pone.0061991.s003.tif]
