# Supplementary material for: Acidosis Activation of the Proton-Sensing GPR4 Receptor Stimulates Vascular Endothelial Cell Inflammatory Responses Revealed by Transcriptome Analysis
Source: PLoS One. 2013 Apr 16;8(4):e61991. doi: 10.1371/journal.pone.0061991 (PMC3628782; doi:10.1371/journal.pone.0061991)
Supplement: Table S1 — A list of TaqMan pre-designed primer-probes used in the study. (DOC) [file pone.0061991.s004.doc]

Supplementary Table S1. A list of TaqMan pre-designed primer-probes used in the study.

| **Gene Symbol** | **Assay ID** |
| --- | --- |
| 18S rRNA | Hs99999901_s1 |
| ACTB | Hs99999903_m1 |
| CCL20 | Hs00355476_m1 |
| CD69 | Hs00934033_m1 |
| CXCL2 | Hs00601975_m1 |
|  | Hs00236966_m1 |
| EGR1 | Hs00152928_m1 |
| EGR2 | Hs00166165_m1 |
| EGR3 | Hs00231780_m1 |
| GAPDH | Hs99999905_m1 |
| ICAM1 | Hs00164932_m1 |
|  | Hs99999152_m1 |
| IL1A | Hs99999028_m1 |
| IL8 | Hs00174103_m1 |
| NFKIBZ | Hs00230071_m1 |
| PTGS2 | Hs00153133_m1 |
| RELB | Hs00232399_m1 |
| SELE | Hs00174057_m1 |
|  | Hs00950401_m1 |
| SELP | Hs00174583_m1 |
| SLC2A1 | Hs00892681_m1 |
| TNFRSF9 | Hs00155512_m1 |
| TRAF1 | Hs01090170_m1 |
| VCAM1 | Hs01003372_m1 |
| VEGFA | Hs00900055_m1 |
