# Supplementary material for: Acidosis Activation of the Proton-Sensing GPR4 Receptor Stimulates Vascular Endothelial Cell Inflammatory Responses Revealed by Transcriptome Analysis
Source: PLoS One. 2013 Apr 16;8(4):e61991. doi: 10.1371/journal.pone.0061991 (PMC3628782; doi:10.1371/journal.pone.0061991)
Supplement: Table S7 — Fold changes of gene expression by real-time RT-PCR in HUVEC/Vector and HUVEC/GPR4 cells upon varying pH treatment. (DOC) [file pone.0061991.s010.doc]

Supplementary Table S7. Fold changes of gene expression by real-time RT-PCR in HUVEC/Vector and HUVEC/GPR4 cells upon varying pH treatment.

| **Gene Symbol** | **HUVEC/Vector** | | | **HUVEC/GPR4** | | |
| --- | --- | --- | --- | --- | --- | --- |
|  | **pH 8.4** | **pH 7.4** | **pH 6.4** | **pH 8.4** | **pH 7.4** | **pH 6.4** |
| CXCL2 | **1.00**±0.03 | **1.46**±0.10 | **3.77**±0.52 | **1.00**±0.03 | **6.30**±1.63 | **189.85**±21.80 |
| CCL20 | **1.00**±0.02 | **1.13**±0.03 | **4.25**±0.93 | **1.01**±0.06 | **8.65**±2.47 | **409.66**±90.13 |
| VCAM1 | **1.02**±0.26 | **1.98**±0.52 | **6.82**±1.67 | **1.03**±0.33 | **4.07**±0.65 | **93.97**±0.20 |
| SELE | **1.00**±0.09 | **1.97**±0.34 | **6.32**±0.89 | **1.03**±0.35 | **1.95**±0.05 | **101.40**±7.50 |
| ICAM1 | **1.00**±0.06 | **0.80**±0.03 | **1.18**±0.13 | **1.00**±0.06 | **2.12**±0.73 | **6.91**±0.34 |
| CD69 | **1.00**±0.02 | **1.27**±0.12 | **2.37**±0.19 | **1.00**±0.04 | **12.46**±0.15 | **41.50**±0.56 |
| IL8 | **1.00**±0.01 | **2.24**±0.37 | **8.39**±1.72 | **1.00**±0.02 | **6.32**±0.69 | **71.38**±5.82 |
| IL1A | **1.00**±0.02 | **1.24**±0.00 | **1.44**±0.03 | **1.00**±0.06 | **2.04**±0.01 | **5.35**±0.11 |
| PTGS2 | **1.00**±0.03 | **0.68**±0.03 | **1.80**±0.20 | **1.00**±0.01 | **0.80**±0.07 | **12.11**±1.29 |
| RELB | **1.00**±0.01 | **1.04** ±0.09 | **1.72**±0.21 | **1.00**±0.02 | **4.48**±0.84 | **10.16**±1.24 |
| TRAF1 | **1.00**±0.02 | **0.96**±0.08 | **1.53**±0.40 | **1.00**±0.02 | **4.35**±1.05 | **23.29**±6.24 |
| EGR1 | **1.01**±0.03 | **2.11**±0.22 | **4.37**±0.65 | **1.00**±0.01 | **1.95**±0.16 | **5.70**±0.66 |
| EGR2 | **1.01**±0.05 | **2.14**±0.24 | **6.32**±1.28 | **1.00**±0.02 | **2.01**±0.20 | **5.08**±0.70 |
| EGR3 | **1.00**±0.03 | **1.23**±0.08 | **2.19**±0.23 | **1.00**±0.01 | **1.03**±0.23 | **5.52**±1.57 |
| DDIT3 | **1.00**±0.02 | **1.24**±0.25 | **3.02**±0.04 | **1.00**±0.14 | **0.97**±0.03 | **7.20**±0.42 |
| FOXF1 | **1.00**±0.01 | **2.02**±0.33 | **2.40**±0.29 | **1.00**±0.12 | **1.33**±0.05 | **3.64**±0.20 |
| ATF3 | **1.00**±0.00 | **0.97**±0.02 | **4.02**±0.11 | **1.00**±0.02 | **1.44**±0.12 | **27.03**±0.91 |
| KLF9 | **1.00**±0.10 | **1.52**±0.05 | **2.55**±0.04 | **1.00**±0.02 | **1.22**±0.17 | **2.73**±0.01 |
